# Efficacy of mucosal polyanhydride nanovaccine against respiratory syncytial virus infection in the neonatal calf

**DOI:** 10.1038/s41598-018-21292-2

**Published:** 2018-02-14

**Authors:** Jodi L. McGill, Sean M. Kelly, Pankaj Kumar, Savannah Speckhart, Shannon L. Haughney, Jamie Henningson, Balaji Narasimhan, Randy E. Sacco

**Affiliations:** 1 Department of Diagnostic Medicine and Pathobiology, Kansas State University, Manhattan, KS USA; 2 Department of Chemical and Biological Engineering, Iowa State University, Ames, IA USA; 3 Ruminant Diseases and Immunology Research Unit, National Animal Disease Center, Agricultural Research Service, USDA, Ames, IA USA; 4 Nanovaccine Institute, Ames, IA USA; 5 Present Address: Charles River Laboratories, Spencerville, OH USA; 6 Present Address: Department of Animal Science, University of Tennessee, Knoxville, TN USA

## Abstract

Human respiratory syncytial virus (HRSV) is a leading cause of severe acute lower respiratory tract infection in infants and children worldwide. Bovine RSV (BRSV) is closely related to HRSV and a significant cause of morbidity in young cattle. BRSV infection in calves displays many similarities to RSV infection in humans, including similar age dependency and disease pathogenesis. Polyanhydride nanoparticle-based vaccines (i.e., nanovaccines) have shown promise as adjuvants and vaccine delivery vehicles due to their ability to promote enhanced immunogenicity through the route of administration, provide sustained antigen exposure, and induce both antibody- and cell-mediated immunity. Here, we developed a novel, mucosal nanovaccine that encapsulates the post-fusion F and G glycoproteins from BRSV into polyanhydride nanoparticles and determined the efficacy of the vaccine against RSV infection using a neonatal calf model. Calves receiving the BRSV-F/G nanovaccine exhibited reduced pathology in the lungs, reduced viral burden, and decreased virus shedding compared to unvaccinated control calves, which correlated with BRSV-specific immune responses in the respiratory tract and peripheral blood. Our results indicate that the BRSV-F/G nanovaccine is highly immunogenic and, with optimization, has the potential to significantly reduce the disease burden associated with RSV infection in both humans and animals.

## Introduction

Human respiratory syncytial virus (HRSV) is a leading cause of severe acute lower respiratory tract disease in infants and young children worldwide^1^ and accounts for up to 70% of hospitalized bronchiolitis cases in industrialized countries^2^. Globally, there are an estimated 33 million new episodes of HRSV-associated disease in children under five years of age with more than 100,000 resultant deaths^3^. Severe RSV infection has been linked with the development and exacerbation of recurrent wheezing and asthma^4^, and is a predisposing factor to the development of otitis media^5^. To date, there are no safe and effective vaccines available for HRSV.

RSV-specific immune responses are directed against a number of viral proteins; however, the fusion (F) and attachment (G) proteins appear to be the most important targets^6^; and thus represent attractive options for the development of subunit vaccines. The F protein exists in two forms on the virion surface: a metastable pre-fusion form and a stable post-fusion trimer. Post-fusion F contains two major neutralizing epitopes, antigenic sites II and IV^7^. Vaccines utilizing post-fusion F elicit high-affinity, site-II directed neutralizing antibodies and have been shown to be highly efficacious in mice and cotton rats^8,9^. Post-fusion F is also highly stable, making it an appealing candidate for incorporation into a subunit vaccine. Reports in mice have shown that vaccines that elicit G-protein specific antibody and T cell responses are protective against RSV infection^10,11^. Bastien *et al*. also demonstrated that immunization with a conserved peptide from the BRSV G protein afforded partial protection against BRSV infection in cattle^11^. The F and G proteins have also been shown to be suitable vaccine targets in cattle, as recombinant vector-vaccines targeting the BRSV F^12,13^ and G^13–15^ proteins, and recombinant plasmid-based strategies targeting the G protein^15,16^ have both been shown to reduce virus shedding and BRSV-associated lung pathology in challenged calves.

A significant barrier to the development of a safe and efficacious vaccine for use against HRSV has been the lack of a suitable animal model. Although mice and cotton rats are commonly used to test candidate vaccines, they are only semi-permissive for HRSV replication, and the pathogenesis of disease significantly differs from that observed in human infants (recently reviewed in^17^). Bovine respiratory syncytial virus (BRSV) is genetically and antigenically closely related to HRSV and is a primary cause of severe acute lower respiratory tract disease in young cattle. Although host specific, BRSV and HRSV display similar epidemiology, pathogenesis and clinical expression in their respective hosts. Development of an efficacious vaccine for BRSV poses similar challenges to that faced for HRSV, particularly the need to immunize the very young, the presence of maternally-derived antibodies and the possibility for the development of vaccine-enhanced disease. Thus, the calf is an ideal model for testing novel vaccine candidates and determining appropriate correlates of vaccine-induced protection from infection.

Polyanhydride nanoparticle-based vaccines (i.e., nanovaccines) have been studied extensively as a vaccine adjuvant and/or delivery platform and provide a safe, efficacious alternative to traditional vaccines^18^. These biodegradable polymers are based on 1,6-bis(*p*-carboxyphenoxy)hexane (CPH), 1,8-bis(*p*-carboxyphenoxy)-3,6-dioxaoctane (CPTEG), and sebacic anhydride (SA). They are biocompatible^19,20^, have been shown to stabilize labile proteins^21–23^, and demonstrate sustained release of encapsulated proteins due to their surface-erodible properties^22,24–26^, allowing for the possibility of single-dose administration. In addition, polyanhydride nanovaccines display chemistry-dependent cellular uptake and intracellular persistence by antigen presenting cells (APC)^27–29^, which can aid in adjuvanting poorly immunogenic proteins. These nanovaccines can be delivered using multiple routes of administration, including intranasally (i.n.)^26,29–31^. Finally, these nanoadjuvants have been shown to induce sustained humoral^32^ and cellular immunity^33,34^. These properties have been exploited to design nanovaccine formulations against multiple pathogens, including *Yersinia pestis*, *Bacillus anthracis*, *Streptococcus pneumoniae*, and influenza A virus^18^. The nanovaccine chosen for the current studies is based on the amphiphilic 50:50 CPTEG:CPH due to its enhanced pathogen-mimicking characteristics^29^, improved stability of encapsulated antigen^21,22^, and suitability as an effective formulation for i.n. delivery^26,29–31^.

Here, we report the development of a polyanhydride-based nanovaccine that contains the recombinant post-fusion F and G proteins from BRSV and demonstrate for the first time, that a single, i.n. immunization of the BRSV-F/G nanovaccine induces robust local cellular and humoral immunity in the respiratory tract, reduced virus-associated pathology, reduced viral burden, and decreased incidence of virus shedding in neonatal calves.

## Results

### *In vitro* release kinetics of BRSV-F/G nanoparticles

We produced a recombinant form of the post-fusion F protein from BRSV strain 375 using a baculovirus expression system; and a recombinant form of the G protein from BRSV strain 375 using an *E. coli* expression system as described in Materials and Methods. The recombinant BRSV F and G proteins were then co-encapsulated at 2.4% by weight into 50:50 CPTEG:CPH nanoparticles.

**Figure 1:**
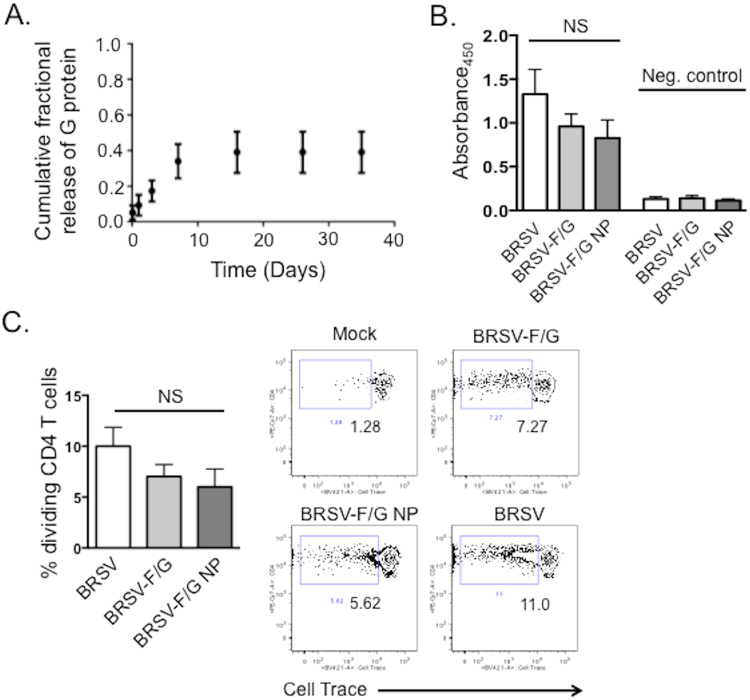
Recombinant BRSV F and G proteins are stable and immunogenic following encapsulation and release from polyanhydride nanoparticles. (**A**) G protein release kinetics from polyanhydride nanoparticles. Data shown is the cumulative mass fraction of G protein released from 50:50 CPTEG:CPH nanoparticles. Data represent means ± SEM. Results are representative of three independent experiments with duplicate samples used in each experiment. (**B**) ELISA plates were coated with 5 μg/mL (2.5 μg/mL each) of the recombinant F and G proteins (BRSV-F/G), or with 100 μL/well of BRSV virus stock (~10^4^ TCID_50_) grown in BT cells. Recombinant BRSV F and G proteins were encapsulated in 50:50 CPTEG:CPH particles and released as described. The released proteins were also coated onto ELISA plates at ~5 μg/mL (BRSV-F/G NP). Sera from BRSV-immune cows were diluted 1:1000 and added to the plates. The binding of bovine IgG to the virus or recombinant proteins was measured by absorbance. Sera were also collected from 2 colostrum-deprived calves, diluted 1:1000, and included as negative control samples. (**C**) PBMC were labeled with Cell Trace Violet and stimulated for 6 days with 5 μg/mL of the recombinant BRSV F and G proteins; 5 μg/mL recombinant BRSV F and G proteins that were encapsulated and released from the CPTEG:CPH particles; or 0.01 MOI of BRSV strain 375. Pokeweed Mitogen was used at a concentration of 1 μg/mL as a positive control. Mock stimulated samples (negative control wells) were cultured with cRPMI and were used to correct for background proliferation. After 6 days, antigen-specific CD4 T cell proliferation was assessed by flow cytometry. Representative flow plots and aggregate results are shown in (**C**). Background levels of proliferation were subtracted and results are presented as change over mock. (**B**,**C**) Results are pooled from 2–3 independent experiments for a total of n = 8–10 animals. Data represent means ± SEM. *p < 0.05 **p < 0.01 NS: not significant compared to BRSV.

The release kinetics of the BRSV-F/G nanovaccine was simulated by using 2% G protein-loaded 50:50 CPTEG:CPH nanoparticles (Fig. 1A). The data show that approximately 40% of G protein encapsulated within the nanoparticles was released within 35 days. This amphiphilic formulation provided zero-order sustained release of the immunogen over the first seven days, followed by a slower rate of release beyond day seven. The encapsulation efficiency of G protein in the nanovaccine was ca. 20%. The G protein release kinetics are consistent with previous observations of protein release kinetics from polyanhydride nanoparticles^21,22,25,26,35,36^.

### Immunogenicity of recombinant BRSV-F/G is preserved following release from CPTEG:CPH nanoparticles

We next confirmed that the recombinant BRSV F and G proteins were immunogenic and stable following encapsulation and release from the 50:50 CPTEG:CPH nanovaccine. As shown in Fig. 1B, serum IgG from BRSV-vaccinated cows recognized both BRSV and the recombinant F and G proteins, with no difference in the response to the encapsulated and released F and G proteins. Serum from the colostrum-deprived animals did not react with BRSV or the recombinant proteins. As shown in Fig. 1C, CD4 T cells from BRSV-immune cows underwent clonal expansion in recall response to BRSV, recombinant BRSV-F/G and the released BRSV-F/G proteins. We observed no significant differences in the response to the encapsulated and released proteins compared to the unencapsulated recombinant proteins.

### Amphiphilic CPTEG:CPH particles activate bovine monocyte-derived dendritic cells (moDC)

**Figure 2:**
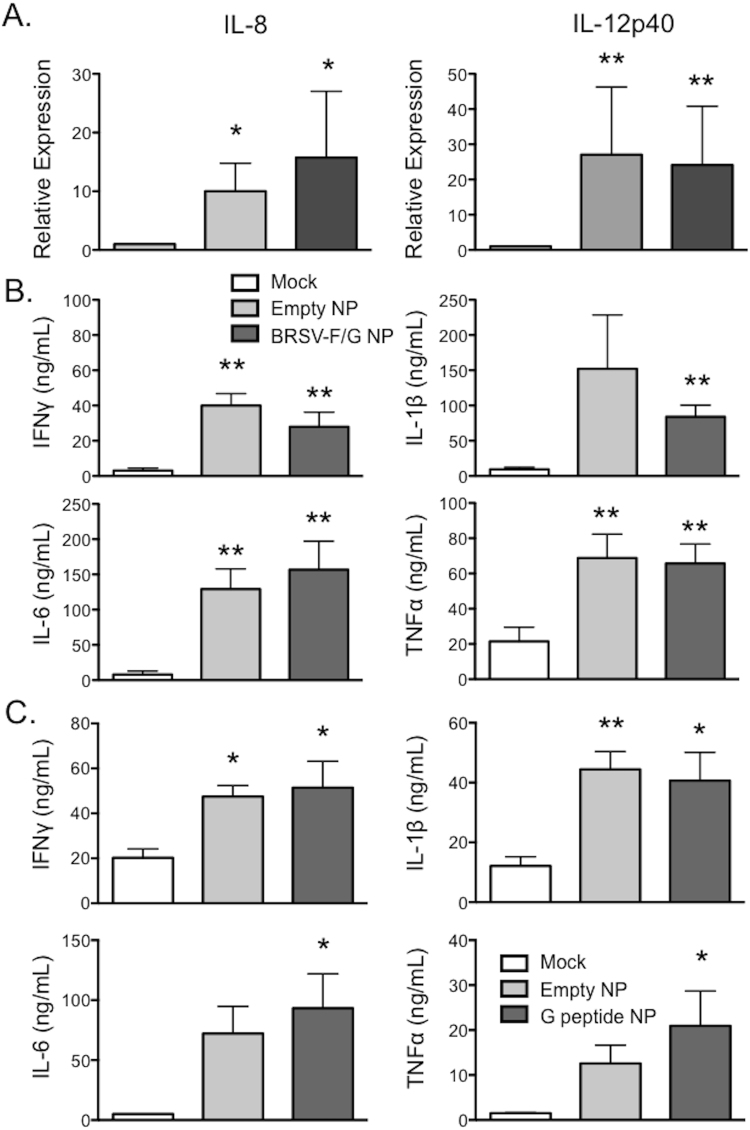
Polyanhydride particles activate bovine APC. Bovine moDC were seeded at 5 × 10^5^ cells per well in a 24-well plate and stimulated with 10 μg/mL ‘empty’ or BRSV-F/G-loaded 50:50 CPTEG:CPH particles. Mock cultures were treated with media only. (**A**) After 18 hours, RNA was isolated from the cells and analyzed by qPCR for expression of IL-8 and IL-12p40. For qPCR analysis, results were normalized to the housekeeping gene RPS-9, and expressed relative to unstimulated control samples. (**B**) After 48 hours, cell culture supernatants were collected and analyzed by ELISAs for concentrations of IFNγ, IL-6, IL-1β and TNFα. (**C**) Alveolar macrophages were isolated from the BAL fluid of healthy calves and were seeded at a concentration of 5 × 10^5^ cells per well in 24-well plates. The macrophages were stimulated with empty CPTEG:CPH particles or with CPTEG:CPH nanoparticles that were loaded with peptides from the BRSV G protein. Mock cultures were treated with media only. After 48 hours, cell culture supernatants were analyzed by multiplex immunoassay. (**A**–**C**) Results were pooled from two independent experiments for a total of n = 8–10 animals. Data represent means ± SEM. *p < 0.05 **p < 0.01 compared to mock stimulated cultures.

Previous reports have shown that CPTEG:CPH nanoparticles have the capacity to activate murine antigen presenting cells^27,37^. Therefore, we determined if the 50:50 CPTEG:CPH nanovaccine was able to activate bovine APC. MoDC were generated from adult, BRSV-immune cows. The cells were stimulated with 10 μg/mL of ‘empty’ or BRSV-F/G-loaded CPTEG:CPH nanoparticles and expression of IL-8 and IL-12p40 was assessed by qPCR, and inflammatory cytokine production was assessed by ELISA. Consistent with reports from mice, the nanovaccine induced moDC activation, as measured by increased expression of IL-8 and IL-12p40 (Fig. 2A) and increased production of IFNγ, IL-6, IL-1β and TNFα (Fig. 2B). In Fig. 2C, alveolar macrophages were stimulated with 10 μg/mL CPTEG:CPH nanoparticles that were ‘empty’, as in Fig. 2A,B, or were stimulated with CPTEG:CPH nanoparticles that were loaded with overlapping 20-mer peptides from the BRSV G protein. Inflammatory cytokine production was measured by multiplex immunoassay. Consistent with our results in Fig. 2A,B, we observed increased inflammatory cytokine expression by nanovaccine-stimulated alveolar macrophages compared to untreated controls, and observed no difference in the response to ‘empty’ or peptide-loaded nanoparticles (Fig. 2C). In separate experiments, we also determined the capacity of the G-peptide loaded nanoparticles to activate moDC. Consistent with our results in Fig. 2, moDC increased inflammatory cytokine production (data not shown) and upregulated surface expression of costimulatory molecules (Supplementary Figure 1) in response to CPTEG:CPH nanoparticle treatment, and we observed no differences between the ‘empty’ and G-peptide loaded nanoparticles. Together our results confirm that the CPTEG:CPH nanoparticles are able to activate bovine APC and demonstrate that this response is largely independent of the antigen payload, as ‘empty’ particles, G peptide-loaded particles, and particles loaded with recombinant F and G proteins elicit similar inflammatory responses.

### *In vivo* immunogenicity and efficacy of the BRSV-F/G nanovaccine

To determine the efficacy of the BRSV-F/G CPTEG:CPH nanovaccine in neonatal calves, animals were vaccinated i.n. with 190 mg ‘empty’ CPTEG:CPH nanoparticles or 190 mg BRSV-F/G-loaded nanoparticles. Four weeks later, calves were challenged via aerosol inoculation with ~10^4^ BRSV strain 375.

Calves were monitored daily for clinical signs and rectal temperatures. Several animals in the unvaccinated group demonstrated elevated temperatures (40–41 °C) for 1–2 days during infection; however, the fevers were not prolonged and no significant differences in body temperature were observed between vaccinated and unvaccinated groups. Mild clinical signs were observed in the BRSV challenged animals, including coughing, increased respiration rates and increased expiratory effort. Clinical signs of experimental BRSV infection were apparent starting on days 4–6 after infection and were recorded in at least some animals from each group. Mild clinical signs were observed in 4/6 calves in the unvaccinated control group, 3/6 calves in the ‘empty’ nanovaccine-administered groups and 2/6 calves in the BRSV-F/G nanovaccine administered group. However, due to variability between animals and disease kinetics, the clinical scores did not differ significantly between vaccinated and unvaccinated groups. No clinical signs were observed in the uninfected control animals at any time during the study.

### Reduced gross and microscopic pathology in BRSV-F/G nanovaccine-administered calves

**Figure 3:**
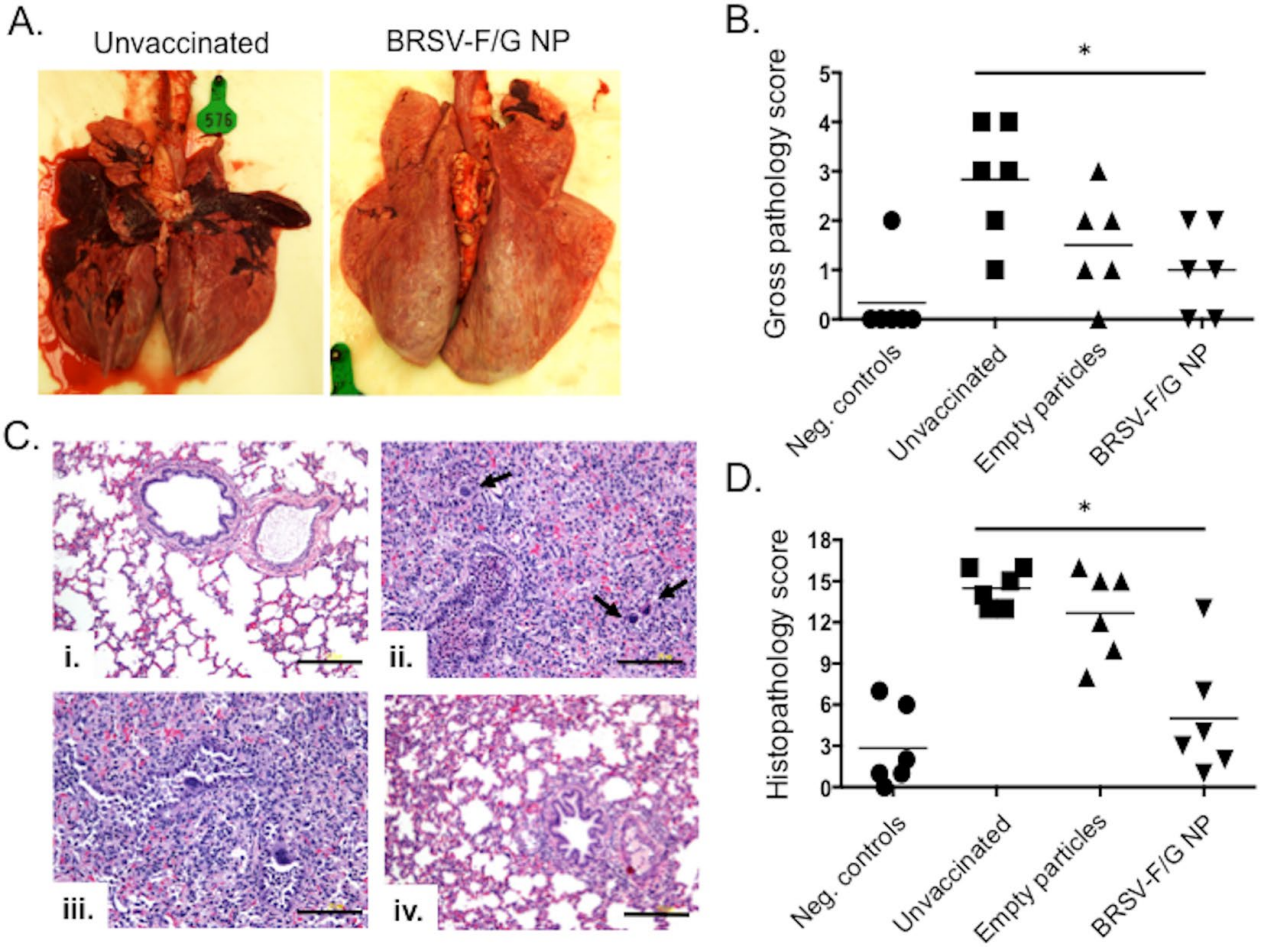
Reduced BRSV-associated gross and microscopic pathology in BRSV-F/G nanovaccine-administered calves. Treatment groups included unvaccinated, uninfected negative control calves; unvaccinated calves challenged with BRSV strain 375; calves vaccinated with ‘empty’ nanoparticles and challenged with BRSV strain 375; and calves vaccinated with the BRSV-F/G nanovaccine and challenged with BRSV strain 375. Animals were euthanized and necropsied on day 7 after challenge. The extent of gross pneumonic consolidation was evaluated based upon the percent of lung affected: a score of 0 was given to lungs free of lesions; 1 was given to lungs with 1–5% affected; 2 was given for 5–15% affected; 3 with 15–30% affected; 4 to lungs with 30–50% of consolidated tissue; and 5 for lungs >50% affected. Representative images from an unvaccinated, infected calf and a calf which received the BRSV-F/G nanovaccine are depicted in (**A**). Aggregate gross pathology results from all groups and all animals are presented in (**B**). (**C**) Sections of lung were collected from multiple locations and microscopic lesions were evaluated by a pathologist in a blinded manner. The severity of the lung lesions was scored based upon six criteria as outlined in Supplementary Table 2. Representative histological images from each of the four groups of calves: uninfected control calf (i), unvaccinated calf (ii), an empty nanovaccinated control calf (iii) and a BRSV nanovaccine-administered calf (iv). Hematoxylin and eosin stain. (i) and (iv)- Normal lung architecture. Note bronchioles are lined by normal tall columnar epithelium and there is no inflammation in the airways. (ii) and (iii)- Severe bronchointerstitial pneumonia with necrotizing bronchiolitis. Bronchioles are filled with degenerate neutrophils, sloughed epithelial cells and necrotic cell debris. (ii) The bronchiolar epithelial cells occasionally form multinucleated syncytia cells (arrows). Alveoli contain variable combinations of macrophages, syncytial cells with foamy vacuolated cytoplasm, lymphocytes and lesser numbers of neutrophils. (iii) The affected bronchioles show syncytia within the lining epithelium and some inflammatory cells mixed with desquamated cells within the lumen. (i and iv) Black scale bars represent 200 μm. (ii and iii) Black scale bars represent 100 μm. Aggregate microscopic pathology results are presented in (**D**). Results represent n = 6 animals/group. The lines indicate the means of each group. *p < 0.05 compared to unvaccinated control calves.

Animals were euthanized on day 7 p.i. We observed gross lesions in these animals that were consistent with our previous reports^38^. The lesions were bilateral and most frequently observed in the cranioventral lung lobes, consisting of multifocal to coalescing areas of firm, pneumonic consolidation (Fig. 3A). The extent of gross pathology in the lungs was evaluated using the criteria outlined in Supplementary Table 1. Unvaccinated control animals developed large, diffuse gross lesions, affecting as much as 40–50% of the lung, while calves receiving the BRSV nanovaccine demonstrated fewer lesions and a significant reduction in the area of lung affected (Fig. 3B).

Representative micrographs from an uninfected control calf (i), an unvaccinated positive control calf (ii), an ‘empty’ nanovaccine-administered calf (iii) and a BRSV-F/G vaccinated calf (iv) are shown in Fig. 3C. Cumulative histopathology scores are depicted in Fig. 3D. The scores for the individual histopathological categories are presented in Supplementary Figure 2. Pulmonary lesions were most pronounced in the unvaccinated control animals and included thickened alveolar septa with infiltrates of macrophages, lymphocytes and occasional neutrophils; and bronchioles filled with neutrophils, sloughed epithelial cells and necrotic cell debris. Five of the six BRSV-F/G nanovaccine-administered calves exhibited reduced histological lesions compared to the unvaccinated calves, with only mild peribronchiolar lymphocytic infiltration and minor accumulation of alveolar exudates. We observed no evidence that the BRSV-F/G nanovaccine promoted the development of enhanced or exacerbated disease.

### Reduced viral burden and reduced virus shedding in BRSV-F/G nanovaccine-administered calves

Nasal swabs and lung tissues were assessed for virus isolation. BRSV was isolated from 5/6 unvaccinated calves on day 3 p.i., and 6/6 animals on day 6 p.i.. Virus was isolated from the lungs of all 6 unvaccinated controls at necropsy. In contrast, BRSV was isolated from only 2/6 BRSV-F/G nanovaccine-administered calves on day 3, and 1/6 on day 6 p.i. Virus was isolated from the lungs of this same animal on day 7 p.i. BRSV was isolated from the nasal swabs of 4/6 calves in the ‘empty’ nanovaccine-administered group on days 3 and 6 after infection, and from the lung tissues of 3/6 calves on day 7 p.i. We did not isolate virus from the nasal swabs collected from any of the animals on day 0 (prior to BRSV challenge), nor did we isolate BRSV from the nasal swabs or lung tissues of the uninfected control calves.

**Figure 4:**
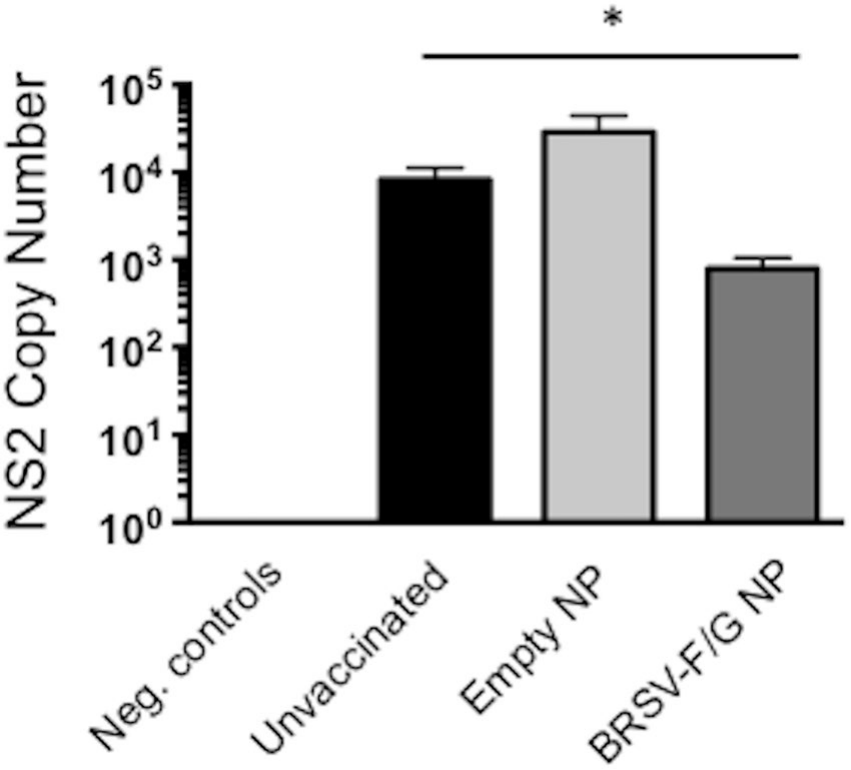
Reduced viral burden in the lungs of BRSV-F/G nanovaccine-administered calves. Treatment groups are outlined in Fig. 3. Samples were collected from 2–3 representative lesion and non-lesion sites of the lungs on day 7 post-challenged and preserved in RNALater. The RNA was extracted using Trizol reagent. The RNA from multiple sites was then pooled and was analyzed by qPCR for the BRSV NS2 gene. Viral NS2 copy numbers were calculated using standard curves and normalized to the housekeeping gene, S9, to correct for differences in input material. Results represent the mean NS2 copy number of each calf, with n = 6 animals/group. The graph depicts means ± SEM of each group. *p < 0.05 compared to unvaccinated control calves.

We performed qPCR for the BRSV NS2 gene on lung tissues collected on day 7 p.i. Consistent with our virus isolation results, BRSV-F/G nanovaccine-administered calves demonstrated significantly reduced quantities of viral RNA compared to their unvaccinated cohorts (Fig. 4).

### BRSV-specific IgA in the nasal and BAL fluid of BRSV-F/G nanovaccine-administered calves

**Figure 5:**
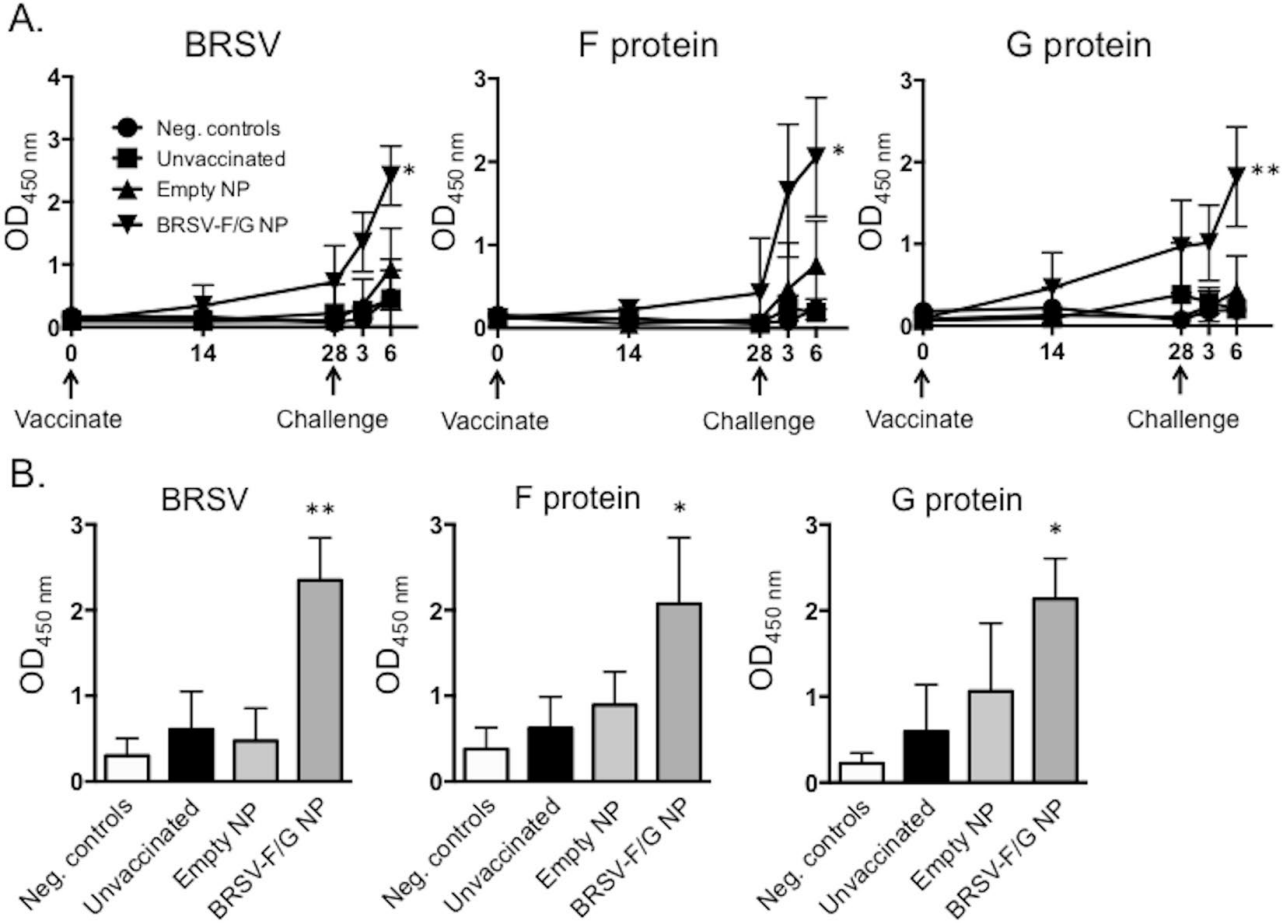
Increased BRSV-specific IgA in the nasal fluid and BAL fluid of BRSV-F/G nanovaccine-administered calves. Treatment groups are outlined in Fig. 3. Nasal fluid was collected on days 0, 14 and 28 post-vaccination, and on days 3 and 6 post-challenge. BAL fluid was collected during necropsy on day 7 post-challenge. The samples were diluted 1:2. Indirect ELISAs were used to quantify BRSV-, F-protein or G-protein specific IgA in (**A**) nasal fluid and (**B**) BAL fluid. Results represent n = 6 animals/group. Data represent means ± SEM. *p < 0.05 **p < 0.01 compared to unvaccinated control calves.

Nasal fluid samples were assessed for BRSV-, F- and G-specific IgA following vaccination and challenge. We observed no significant changes in BRSV-specific IgA on day 28 after vaccination. By day 6 after infection, BRSV-F/G nanovaccine-administered calves demonstrated a significant increase in virus-specific IgA compared to the unvaccinated calves or animals receiving the ‘empty’ nanovaccine (Fig. 5A). We also observed increased BRSV-, F- and G-specific IgA in the BAL fluid from BRSV-F/G nanovaccine-administered calves by day 7 after infection (Fig. 5B).

We performed virus-neutralization assays using nasal fluid to determine if neutralizing antibody responses were generated in the respiratory tract. On day 28-post vaccination (day 0 prior to challenge), we measured low titers of neutralizing antibody in the nasal fluid of all animals, with no significant differences observed between treatment groups (Table 1). By day 6 after infection, we observed an increase in neutralizing antibody titers in the nasal fluid of all BRSV-challenged groups, and noted significantly higher titers (p ≤ 0.01) in the nasal fluid of BRSV-F/G nanovaccine-administered animals compared with the unvaccinated control animals. Importantly, these results indicate that a single dose of the BRSV-F/G nanovaccine promotes significant production of neutralizing, BRSV-specific antibodies in the respiratory tract of neonatal calves.

**Table 1:** Virus neutralization titers measured in the nasal fluid on day 28 (day 0 before challenge) and day 6 post-challenge.

Group	Day 28 (challenge day) mean neutralization titer (range)	Day 6 mean neutralization titer (range)
Negative controls	5 (2–8)	4 (2–8)
Unvaccinated	4.6 (2–8)	20 (8–32)
Empty nanovax	5.3 (4–8)	34.6 (16–64)
BRSV-F/G nanovax	20 (8–32)	108 (64–256)**

Data are presented as the mean (range) titer per group. **p < 0.01 compared to unvaccinated control animals.

Given the importance of developing an RSV vaccine that is efficacious in the face of maternal antibody, we selected colostrum-replete animals for these studies. Calves in all groups demonstrated pre-existing BRSV-specific IgG in the serum, with neutralizing antibody titers ranging from 16–256 on the day of vaccination; 16–128 on day 28 post-vaccination (day 0, prior to challenge); and 8–128 on day 6 after challenge. We observed no significant differences between treatment groups, nor did we observe any consistent changes in serum neutralizing titers related to BRSV-F/G nanovaccine-administration or BRSV challenge.

### BRSV-specific cellular responses in the lungs and peripheral blood of BRSV-F/G nanovaccine-administered calves

**Figure 6:**
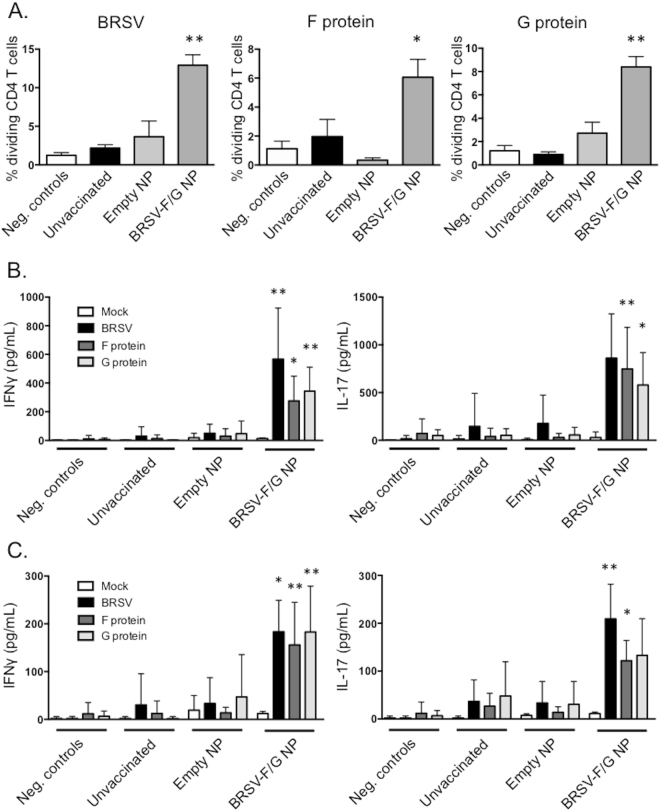
Enhanced BRSV-specific T cell responses in the peripheral blood and BAL of BRSV-F/G nanovaccine-administered calves. PBMC were collected on day 6 post-challenge, labeled with Cell Trace Violet and stimulated for 6 days with 5 μg/mL of the recombinant BRSV F and G proteins, or 0.01 MOI of BRSV strain 375. Pokeweed Mitogen was used at a concentration of 1 μg/mL as a positive control. Mock stimulated samples (negative control wells) were cultured with cRPMI and were used to correct for background proliferation. (**A**) After 6 days, antigen-specific CD4 T cell proliferation was assessed by flow cytometry, as measured by dilution of the Cell Trace Violet dye. Background levels of proliferation were subtracted and results are presented as change over mock. (**B**) Stimulated cell culture supernatants were collected after 6 days and concentrations of IFNγ (left panel) and IL-17A (right panel) were measured by commercial sandwich ELISAs. (**C**) BALs were performed on day 7 post-challenge. Cells were enumerated and stimulated for 6 days with recombinant BRSV F protein, G protein or BRSV as in A. After 6 days, cell culture supernatants were collected and concentrations of IFNγ (left panel) and IL-17A (right panel) were measured by commercial sandwich ELISAs. Results represent n = 6 animals/group. Data represent means ± SEM. *p < 0.05 **p < 0.01 compared to unvaccinated controls.

Prior to challenge, we detected no significant BRSV-, F- and G-protein specific CD4 or CD8 T cell proliferation in PBMCs from any groups. By day 6 p.i., we measured significant antigen-specific proliferation by CD4 T cells from BRSV-F/G nanovaccine-administered calves in response to both live virus and the recombinant proteins (Fig. 6A). PBMCs from the BRSV-F/G nanovaccine-administrated animals also secreted IFNγ and IL-17A in response to whole virus and the recombinant proteins (Fig. 6B). Levels of IL-4 were below the limit of detection for all groups (data not shown).

Mononuclear cells were isolated from the BAL on day 7 p.i. and stimulated with whole virus, F or G protein as in Fig. 6A. We measured a significant increase in the concentration of IFNγ and IL-17 in the BAL cell culture supernatants from the BRSV-F/G nanovaccine-administered calves, but not control calves, in response to protein or whole virus stimulation (Fig. 6C).

## Discussion

RSV infection has a devastating, worldwide impact on human health and despite significant efforts, no approved vaccine currently exists for use against the disease in humans. Similarly, although vaccines have been widely available for BRSV for decades, their efficacy in the field is problematic, and BRSV infection in calves continues to have significant impacts on animal health and the agricultural economy^39,40^. The two diseases, HRSV infection in humans and BRSV infection in calves, present with striking similarities in pathogenesis and host immunity (recently reviewed in^17^). Thus, the calf model represents authentic host-pathogen interaction that faithfully replicates many aspects of human RSV infection, and is therefore an ideal model for testing novel vaccine candidates and determining appropriate correlates of vaccine-induced protection. Here, we vaccinated animals that were less than one month of age and had BRSV-specific maternally-derived antibodies. A single, i.n. administration of the BRSV-F/G nanovaccine induced mucosal, anti-viral immunity and protected most animals from virulent BRSV challenge. Calves receiving the BRSV-F/G nanovaccine mounted cellular and humoral immune responses in the upper and lower respiratory tract (Figs 5 and 6, and Table 1); exhibited a reduced viral burden in the lungs on day 7 post challenge (Fig. 4); and developed significantly fewer gross and microscopic lesions in the lungs compared to unvaccinated control animals (Fig. 3).

Our amphiphilic, polyanhydride nanovaccine platform, which is based upon CPTEG:CPH copolymers, offers a number of advantages over other polymeric nanoparticle systems, including: inherent adjuvant properties^27,29,37,41^; the tendency of the particles to surface erode^42^, thus stabilizing the encapsulated proteins and maintaining the structural and biological features of the antigens for longer periods of time^21,22,35,36^; the ability to provide sustained and tunable antigen release kinetics^25^; and the ability to degrade at a neutral pH, into non-toxic and non-mutagenic carboxylic acids^43^. Consistent with previous reports showing that polyanhydride nanoparticles can protect labile proteins from degradation^21–23,35,36,43^, we demonstrated here that the *in vitro* antigenicity of the BRSV F and G proteins was preserved following encapsulation and release from the 50:50 CPTEG:CPH particles (Fig. 1). We observed no significant changes in the capacity of BRSV-specific polyclonal antibodies or BRSV-specific CD4 T cells to recognize the recombinant proteins following their release from the nanoparticles, which is critical for vaccine efficacy. The length of antigen exposure is a vital factor dictating the establishment of long-term immunity, and consistent with prior reports, the CPTEG:CPH nanoparticles used here provided sustained antigen release of the BRSV G protein, with only ~40% of the recombinant protein released by 30 days *in vitro*. In agreement with our *in vitro* findings suggesting that the BRSV-F/G nanovaccine should be stable and immunogenic, our *in vivo* vaccine experiments demonstrated that a single i.n. vaccination, containing a suboptimal dose of antigen, was sufficient to protect a majority of calves from severe BRSV infection.

Both neutralizing IgG and IgA are thought to have a role in protection from RSV. However, we still have a poor understanding of the immune responses that are most important for protection from RSV disease in the neonate, and even less appreciation for the role of maternal antibody in protection from infection. Some studies suggest that maternal antibody can prevent RSV infection^44,45^; and systemic, prophylactic administration of Palivizumab, a monoclonal antibody that is specific to the RSV F protein, is effective at reducing RSV-related hospitalization in high-risk infants^46^. However, both human infants^47–49^ and calves^50^ with maternal antibodies still develop severe RSV disease; and in infants, there is no correlation between titers of virus-specific maternal IgG and prevention of hospitalization with RSV^49,51^. In agreement, despite significant titers of maternally-derived serum antibodies, our unvaccinated control animals developed BRSV disease, including transient fevers, clinical signs, severe pathology in the lungs and significant viral shedding.

Adults who have been repeatedly infected with RSV develop sustained high levels of IgA in nasal secretions, which has been shown to prevent virus replication in the upper airways, regardless of serum Ig levels^52,53^. Mucosal IgA also plays an important role in reducing the occurrence and severity of RSV infection in infants and children^54^. Our results reinforce this conclusion, demonstrating that BRSV-F/G vaccinated calves mounted a significant IgA response in the nasal cavity and BAL that increased rapidly in the days following virulent BRSV challenge. However, in adults, IgA responses can wane^53^ and the IgG response may be more important in long-term protection. We chose to use calves with BRSV-specific maternal antibodies, as it is the most stringent and physiologic model in which to test our novel vaccine platform. We were unable to measure any changes in BRSV serum neutralizing titers following administration of the BRSV-F/G nanovaccine, or following virulent BRSV challenge. Thus, it is currently unknown if a single administration of the BRSV-F/G nanovaccine induces systemic antibody responses. In the future, it may be necessary to dissect the contributions of systemic vs. mucosal vaccine-induced immunity in calves with low or no BRSV-specific maternal antibodies.

We observed a significant rise in neutralizing antibody titers in the nasal secretions of the BRSV-F/G nanovaccine administered calves (Table 1). Based upon the results of our ELISA analysis, this response is presumably mediated by neutralizing IgA. Interestingly, however, we also observed a rapid increase (nearly 4-fold) in neutralizing titer in the nasal fluid of the unvaccinated control calves by six days post infection. It is possible that the early neutralizing response we observed in the unvaccinated and ‘empty’ nanovaccine administered calves was mediated by BRSV-specific IgM, which has been previously observed in nasal secretions from children hospitalized with HRSV infection^55^, and was shown to appear more rapidly than HRSV-specific IgA. BRSV infection causes inflammation in the upper respiratory tract, thus the increase in neutralizing antibody titers could also potentially be attributed to increased levels of serum antibodies leaking into the nasal secretions of the unvaccinated and ‘empty’ nanovaccine administered calves.

The RSV F protein is a type I viral fusion protein that is synthesized as a precursor that is proteolytically cleaved by furin into disulfide-linked fragments^7^. It is highly conserved between virus strains, as well as between HRSV and BRSV, demonstrating approximately 80% homology^56^. The F protein exists in two forms on the virion surface: a metastable pre-fusion form and a stable post-fusion trimer. The post-fusion form of the F protein contains two major neutralizing epitopes, antigenic sites II and IV^7^. High-affinity, site-II directed neutralizing antibodies are protective in mice and cotton rats^8,9^. When considering the design of an F protein based subunit vaccine, protein stability is of paramount concern, and the post-fusion F is advantageous due to its highly stable nature. The prefusion F protein contains antigenic site ø, and recent evidence suggests that this epitope is the primary target for neutralizing antibodies in humans^57^. Vaccine formulations incorporating the pre-fusion form of the F protein are highly efficacious in rodent models^58–61^; and a highly efficacious prefusion F vaccine was also recently reported for BRSV^56^, suggesting that the antigenic ø site is conserved and of immunologic relevance to the calf model as well. Stable expression of the prefusion F protein is not trivial and hurdles exist with respect to affordable, consistent production of sufficient quantities of stable, prefusion F for use in subunit vaccines. However, the availability of the crystal structure^56,62^, and improved strategies to express and stabilize the prefusion F^56,58,61^, significantly enhance the feasibility of a subunit based prefusion F vaccine. In the future, we plan to examine the stability and efficacy of a mucosal, prefusion F-based nanovaccine in neonates.

In summary, we have shown here that a single, intranasal administration of the BRSV-F/G nanovaccine elicits mucosal and systemic antiviral immunity, resulting in reduced virus-associated pathology and reduced viral burdens in animals vaccinated at ≤one month of age. The results of our *in vivo* efficacy studies warrant further evaluation of the BRSV-F/G nanovaccine for protection against RSV infection in both humans and cattle, and have encouraged us to continue to refine the polyanhydride chemistries and vaccine formulations to optimize their efficacy against RSV infection in neonates.

## Materials and Methods

### Protein production and purification

#### Recombinant F protein

The recombinant fusion (F) protein was produced by Genscript (Piscataway, NJ) using a recombinant baculovirus expression system. The expression scheme was selected based upon previous publications^63–65^. The F protein from BRSV strain 375 (L158-T529, GenBank FJ543092.1) was expressed with a truncation at the -COOH terminus, as described by Wathen *et al*.^64^. The construct was then modified at the 3′ end with the addition of a linker sequence, a GCN4 trimerization domain and a HIS-tag. Sf9 cells were grown in Sf‐900 II SFM Expression Medium (Life Technologies) and maintained in Erlenmeyer flasks at 27 °C in an orbital shaker. One day before transfection, the cells were seeded in 6 wells. On the day of transfection, DNA and Cellfectin II (Life Technologies) were added into the plate with cells ready for transfection. Cells were incubated in Sf‐900 II SFM for 5–7 days at 27 °C before harvest. The supernatant was collected after centrifugation and designated as P1 viral stock. P2 was amplified for later infection. For preparation of the recombinant protein, Sf9 cells were infected with the recombinant P2 virus and incubated for 5–7 days at 27 °C before harvest. Cell pellets were harvested and lysed using Triton-X 100 lysis buffer and the precipitation of cell lysate was dissolved using urea. The target protein was obtained by one‐step purification using a Ni column and 8 M urea-based wash buffer. Fractions were pooled and refolded followed by 0.22 μm filter sterilization. The proteins were analyzed by SDS-PAGE and Western blot by using standard protocols for molecular weight and purity measurements. The primary antibody for Western blot was mouse‐anti‐HIS mAb (GenScript). The protein concentration was determined by Bradford protein assay with BSA as a standard. The protein was confirmed to contain <0.1 EU/μg of endotoxin (LAL Endotoxin Assay Kit, Genscript).

#### Recombinant G protein

Recombinant G protein was expressed with the assistance of the Protein Production Group, COBRE Center for Protein Structure and Function, University of Kansas. The ectodomain of the attachment (G) protein from BRSV strain 375 (S67-I257, GenBank L10925.1) was modified at the 3′ end by a HIS-tag and expressed in *E. coli* using the pTBSG vector as previously described^66^. For production of the recombinant protein, recombinant *E. coli* was grown overnight in LB media containing ampicillin and chloramphenicol. The cells were induced with IPTG for three hours, then cells were pelleted. The cell pellets were lysed in 20 mM Tris buffer (pH 8.0) containing 1% Triton-X 100 and frozen. The cell lysate was sonicated until the suspension was no longer viscous and then the soluble fraction removed by centrifugation. The insoluble fraction (inclusion bodies) was solubilized in urea buffer (6 M urea, 50 mM Tris-HCl (pH 8.0), and 500 mM NaCl). The solubilized fraction was purified by Ni-NTA Affinity Chromatography. The endotoxin levels on the resulting purified fractions were confirmed to be less than 1 EU/μg.

### Polymer synthesis and characterization

CPTEG and CPH monomer synthesis and 50:50 CPTEG:CPH copolymer synthesis using melt polycondensation were performed as described previously^25,67^. Copolymer composition and molecular weight were determined using ^1^H NMR (DXR 400), specifically by end group analysis of NMR spectra. The copolymer molecular weight was determined to be 5,003 g/mol with a molar composition of 48% CPTEG and 52% CPH.

### Nanoparticle synthesis

50:50 CPTEG:CPH nanoparticles encapsulating 2.4% w/w protein, consisting of 1.9% w/w G protein and 0.5% w/w F protein, were synthesized using water/oil/oil nanoprecipitation^68^. Briefly, F and G proteins were dissolved into nanopure water at 178 µg/mL. 50:50 CPTEG:CPH copolymer was dissolved in methylene chloride at 20 mg/mL, and the protein solution was pipetted into the solvent. The mixture was sonicated (30 Hz for 30 s), and poured into a non-solvent (pentane chilled to −10 °C) at a solvent to non-solvent ratio of 1 to 250. The suspension was immediately vacuum filtered to recover nanoparticles encapsulating the proteins, with a yield of ca. 62%. Nanoparticles not encapsulating protein (i.e., “empty” nanoparticles) were synthesized as a control using the same procedure as described above. For preliminary *in vitro* studies, CPTEG:CPH nanoparticles were also generated using a cocktail of overlapping, 20-mer peptides from the BRSV G protein. The peptides were encapsulated at 2% w/w protein using the protocol described above. The G-protein peptide nanoparticles were used for the alveolar macrophage studies in Fig. 2C and the moDC data in Supplementary Figure 1.

### Protein release assay

BRSV-G nanoparticles were incubated in microcentrifuge tubes at a concentration of 20 mg/mL in 0.5 mL of 0.1 M PBS buffer (pH 7.4). Samples were sonicated (30 Hz for 30 s) and placed in a shaker incubator at 37 °C. Samples were centrifuged at 10,000 rcf for 10 min and 0.4 mL of supernatant was removed and replaced with fresh buffer at indicated time points. Aliquots were stored at 4 °C and protein mass released was measured via microbicinchoninic acid (microBCA) analysis at an absorbance of 562 nm. After 35 days, unreleased protein was extracted by 40 mM NaOH using 0.2 mL per microcentrifuge tube, as described^69^. The mass of protein released was determined via microBCA analysis and the encapsulation efficiency, which was defined as the total amount of protein released divided by the initial mass of protein, was calculated.

### Animals

#### BRSV-F/G protein immunogenicity studies

Peripheral blood and sera were collected from eight female adult Holstein cows housed at the Kansas State University Dairy in Manhattan, KS. Animals from this herd have BRSV-specific antibodies and BRSV-specific CD4 T cells^70^.

### Vaccine studies

Twenty-four, colostrum replete, mixed-gender Holstein calves were enrolled at 2–3 weeks of age and were randomly assigned to four treatment groups (n = 6 animals/group): unvaccinated, uninfected controls; unvaccinated and challenged with BRSV strain 375; i.n. vaccination with 190 mg total ‘empty’ CPTEG:CPH nanoparticles, followed by BRSV challenge; i.n. vaccination with 190 mg total BRSV-F/G-loaded CPTEG:CPH nanoparticles, followed by BRSV challenge. Each calf received ~0.9 mg total of the recombinant BRSV-F/G proteins. I.n. vaccines were administered in a volume of 5 mL sterile saline, with 2.5 mL injected into each nostril.

Nasal fluid samples were collected at weekly intervals following vaccination, and on days 2, 4 and 6 post BRSV challenge. Commercial, polyurethane sponges were cut into 1–2 inch squares and autoclaved. Sponges were dampened with 1 mL of sterile saline and then a single square was inserted into the calf’s nostril for 5–10 minutes. The sponges were then removed, placed in a tube and an additional 1 mL of sterile saline was added. Liquid was recovered from each sponge by squeezing in the barrel of a 5 mL syringe. The resulting nasal fluid was then aliquoted and frozen at −80 °C for later use.

All animal procedures were conducted in strict accordance with federal and institutional guidelines and were approved by the Kansas State University Institutional Animal Care and Use Committee.

### BRSV inoculum and aerosol challenge model

BRSV strain 375 was prepared from virus stock re-isolated from the lung of an infected animal and passaged less than 4 times on bovine turbinate (BT) cells. The viral inoculum was determined free of contaminating BVDV by PCR. Calves were inoculated via aerosol challenge with ~10^4^ TCID_50_/mL of BRSV strain 375 as previously described^38^.

### Antigen recall assays

Peripheral blood mononuclear cells (PBMCs) were prepared as previously described^70^. Cells were labeled with Cell Trace Violet per manufacturers instructions (Life Technologies) and 5 × 10^6^ cells/mL were plated in round-bottom 96-well plates with 5 μg/mL recombinant BRSV F or G protein or 0.01 MOI of BRSV. For immunogenicity studies, BRSV-F/G was encapsulated into the CPTEG:CPH particles, then released as described, and used to stimulate PBMCs. Plates were incubated for 6 days at 37 °C in a 5% CO_2_ incubator. Cell culture supernatants were stored at −80 °C. PBMC were resuspended in FACS buffer and stained with antibodies specific to bovine CD3, CD4 and CD8 as previously described^70^. Cells were analyzed using a BD LSR Fortessa X20 and FlowJo Software (Treestar).

### Necropsy and pathological evaluation

Calves were euthanized on day 7 post-infection (p.i.) by barbiturate overdose. Pathological evaluation was performed similar to previous descriptions^38,71^. The extent of gross pneumonic consolidation was evaluated using the scoring system similar to that outlined in^71^, and is shown in Supplementary Table 1.

Bronchoalveolar lavage fluid (BAL) was collected by introducing 500 mL of sterile, ice-cold PBS through the trachea. Samples of affected and unaffected lungs were collected from multiple sites for histopathological evaluation. Tissues were fixed by immersion in 10% neutral buffered formalin and processed by routine paraffin-embedment and sectioning. Five μm sections were stained for hematoxylin and eosin. Microscopic lesions were evaluated by a pathologist (Dr. Kumar) in a blinded manner. The severity of the lung lesions was scored based upon the criteria outlined in Supplementary Table 2.

### *In vitro* analysis of nanoparticle immunogenicity in bovine APC

Monocyte-derived dendritic cells (moDC) were prepared from adult cows housed at the KSU dairy. The moDC were generated using a protocol previously described by Werling *et al*.^72^. Recombinant bovine IL-4 and recombinant bovine GM-CSF were purchased from Kingfisher Biotech, Inc. After 6 days, moDC were seeded at 5 × 10^5^ cells per well in 24-well plates and incubated with 10 μg/mL ‘empty’ or BRSV-F/G loaded CPTEG:CPH particles. Alveolar macrophages were isolated from the BAL fluid of normal calves at the National Animal Disease Center in Ames, IA. Macrophages were seeded at 5 × 10^5^ cells per well in 24-well plates and stimulated as above, using the CPTEG:CPH G-peptide loaded nanoparticles. In both sets of experiments, mock cultures were treated with media only. After 18 hours, the cells were preserved for qPCR analysis. After 48 hours, cell culture supernatants were harvested and stored at −80 °C until later analysis.

### Real-time PCR

RNA isolation, cDNA preparation and qPCR were performed as described^70^. The primers for bovine IL-8 and IL-12p40 have been published^38^. Relative gene expression was determined using the 2^−ΔΔCt^ method^73^, with RPS9 as the reference housekeeping gene.

For quantitation of NS2 copy number, lung samples from a representative gross lesion and non-lesioned tissue from each calf were collected and stored in RNAlater. RNA was isolated from lung tissue samples using Trizol Reagen (Life Technologies). Total RNA was quantified by Nanodrop and 500 ng of total RNA were used in each reaction. cDNA synthesis and quantitative rtPCR reactions were carried out using the Taqman RNA-to-CT 1-step kit (Applied Biosystems) per manufacturers instructions using the following primers and probes: NS2 forward, 5′-GAACGACAGGCCACATTTA-3′; NS2 reverse, 5′-AGGCATTGGAAATGTACCATA-3′; NS2 probe, 5′-/56-FAM/TGAAGCTAT/ZEN/TGCATAAAGTGGGTAGCACA/3IABkFQ/-3′; S9 forward, 5′-GTGAACATCCCGTCCTTCAT-3′; S9 reverse, 5′-TCTTGGCGTTCTTCCTCTTC-3′; S9 probe, 5′-/56-FAM/AAGTCGATG/ZEN/TGCTTCTGCGAGTCC/3IABkFQ/-3′. The reactions were performed on an Agilent MX3000P Real-Time PCR machine with the following cycling conditions: 48 °C hold for 15 minutes; 95 °C hold for 10 minutes; 40 cycles of 95 °C for 15 s, then 60 °C for 1 minute. Standard curves for NS2 and S9 genes were run in parallel with test samples, and all standards and test samples were run in triplicate. Viral NS2 copy numbers were calculated using standard curves and normalized to S9 to correct for differences in input material.

### Virus isolation

Nasal swabs were collected from each calf on days 0, 3 and 6 p.i. and placed in sterile PBS. Virus isolations were performed as previously described^38^.

### ELISAs and multiplex cytokine immunoassay

A multiplex immunoassay (Aushon Biosystems) was used to quantify cytokine secretion in supernatants from bovine alveolar macrophages as previously described^70^.

Bovine IL-17A, IFNγ, IL-6, IL-1β and TNFα VetSet ELISA Development kits were purchased from Kingfisher Biotech, Inc. The bovine IL-4 ELISA kit was purchased from Thermo Fisher Scientific. ELISAs were performed according to kit manufacturer’s instructions.

Indirect ELISAs were used to quantify IgA in the nasal and BAL fluid. Indirect ELISAs were also used to determine the immunogenicity of the BRSV F and G proteins prior to encapsulation, and following release from the CPTEG:CPH nanoparticles. For the IgA quantification, ELISA plates were coated overnight at 4 °C with 3 μg/mL F or G protein, or with 100 μl/well of BRSV stock (~10^4^ TCID_50_). For the immunogenicity studies, the ELISA plates were coated overnight at 4 °C with 5 μg/mL total of the F and G protein (2.5 μg/mL of each), with ~5 μg/mL of the BRSV F and G proteins that had been encapsulated and released from the CPTEG:CPH nanoparticles, or with 100 μl/well of BRSV stock (~10^4^ TCID_50_). Negative control wells were coated with 100 μl/well cell culture media prepared from uninfected BT. Nasal fluid samples were diluted 1:2 and treated with 10 mM dithiothreitol (DTT) for 1 hour at 37 °C prior to performing the ELISAs. BAL samples were diluted 1:2 but were not treated with DTT. Serum samples were diluted 1:1000. All samples were plated in duplicates, incubated for 2 hours at room temperature and then washed. Sheep anti-bovine IgA-HRP (Bethyl Laboratories) was used at 0.5 μg/mL. Sheep anti-bovine IgG-HRP (Bethyl Laboratories) was used at 0.5 μg/mL for the immunogenicity experiments. Plates were developed using Pierce 1-Step Ultra TMP Substrate (ThermoScientific Pierce). The reaction was stopped with the addition of 0.2 M H_2_SO_4_ and plates were read at an optical density of 450 nm and 540 nm using an automated plate reader.

### Virus neutralization assay

BT cells were seeded into 96-well flat-bottomed plates. Nasal fluid samples were serially diluted two-fold in serum-free tissue culture medium. BRSV (100 TCID_50_ units) was added to the diluted samples and incubated for 30 minutes at 37 °C. The tissue culture media was removed from the cell monolayers and replaced with the nasal fluid/virus samples. Each sample was assayed in triplicate. The virus was allowed to infect for 90 minutes at 37 °C with occasional rocking, and then the cell monolayers were washed and returned to complete cell culture medium. The plates were observed for cytopathic effect daily for 10 days. The virus neutralizing antibody titer was recorded as the reciprocal of the last dilution of the fluid that was able to prevent infection in 100% of the triplicate wells.

### Statistics

Statistical analysis was performed using Prism v6.0f software (Graphpad Software, Inc.). The data were analyzed using a Kruskal-Wallis test followed by Dunn’s Multiple Comparisons post test.

### Data availability

The datasets generated during and/or analyzed during the current study are available from the corresponding author on reasonable request.

## Electronic supplementary material

Supplementary Information
