# Supplementary material for: Efficacy of mucosal polyanhydride nanovaccine against respiratory syncytial virus infection in the neonatal calf
Source: Sci Rep. 2018 Feb 14;8:3021. doi: 10.1038/s41598-018-21292-2 (PMC5813012; doi:10.1038/s41598-018-21292-2)
Supplement: Supplementary file 1 — Supplementary Information [file 41598_2018_21292_MOESM1_ESM.doc]

**Efficacy of mucosal polyanhydride nanovaccine against respiratory syncytial virus infection in the neonatal calf**

Jodi L. McGill*^1,4^, Sean M. Kelly^2^, Pankaj Kumar^1,5^, Savannah Speckhart^1,6^, Shannon L. Haughney^2^, Jamie Henningson^1^, Balaji Narasimhan^2,4^, Randy E. Sacco^3,4^

^1^Department of Diagnostic Medicine and Pathobiology, Kansas State University, Manhattan, KS

^2^Department of Chemical and Biological Engineering, Iowa State University, Ames, IA

^3^Ruminant Diseases and Immunology Research Unit, National Animal Disease Center, Agricultural Research Service, USDA, Ames, IA

^4^Nanovaccine Institute, Ames, IA

^5^Current address: Charles River Laboratories, Spencerville, OH

^6^Current address: Department of Animal Science, University of Tennessee, Knoxville, TN

***Corresponding Author**

Jodi McGill

Telephone: 785-532-4602

Fax: 785-532-4039

Email: jlmcgill@vet.k-state

**Supplementary Table 1.** Scoring criteria for gross pathology in the lungs. The extent of pneumonic consolidation was evaluated using the scoring criteria outlined previously ^71^.

| **Gross Pathology Score** | **% of lung affected** |
| --- | --- |
| 0 | Lungs free of lesions |
| 1 | 1-5% affected |
| 2 | 5-15% affected |
| 3 | 15-30% affected |
| 4 | 30-50% affected |
| 5 | <50% affected |

**Supplementary Table 2.** Scoring criteria for microscopic pathology in the lungs. The extent of pneumonic consolidation was evaluated using the following criteria. Each category was assigned a score from 0 (none/minimal) to 3 (severe). Scores for each category were totaled to obtain a total score out of 18. Aggregate scores are presented in Figure 3. Scores for each individual category are presented in Supplementary Figure 2.

| **Score** | **0 (none/minimal)** | **1 (mild)** | **2 (moderate)** | **3 (severe)** |
| --- | --- | --- | --- | --- |
| **Airway epithelial necrosis/ attenuation/disruption** | NONE (Normal tall columnar ciliated epithelium) | Affecting up to 10% of airways | Affecting > 10% and <40% of airways | Affecting more than 40% of airways |
| **Accumulation of necrotic debris and inflammatory leukocytes within the bronchiolar lumen** | No/minimal inflammation | Sparsely scattered inflammatory cells affecting occasional airways | More than a few scattered intraluminal inflammatory aggregates | Bronchioles completely blocked by inflammatory/necrotic debris |
| **Percentage of airways with inflammation** | No/minimal inflammation/ Very few airways affected | Affecting up to 10% of airways | Affecting > 10% and <40% of airways | Affecting more than 40% of airways |
| **Peribronchiolar and perivascular lymphocytic inflammation** | None/minimal | Incomplete, or loosely formed cuffs of 1-2 cell layers | Numerous cuffs, predominantly incomplete and loosely-formed with lesser well formed complete cuffs of 3-4 cell layers | Numerous cuffs, predominantly  well-formed with numerous broad, dense cuffs of > 5 cell layers |
| **Alveolar exudate (inflammatory leukocytes/alveolar macrophages/multinucleate giant cells)** | None/minimal | Alveoli and/or interlobular septal involvement. Affecting up to 10% of lung | Alveoli and/or interlobular septal involvement.  Affecting > 10% and <40% of lung | Alveoli and/or interlobular septal involvement. Affecting > 40% of section |
| **Thickening of alveolar septa/interstitium by inflammatory cells** | Septae typically 1-2, or occasionally 3, nucleated cells wide and absence of inflammatory cells | Scattered inflammatory cells within alveolar walls Affecting < 10% of the section | Focal or multifocal alveolar septal inflammation with regions of moderate thickening of septae. Affecting > 10% and <40% | Coalescing to diffuse alveolar septal inflammation. Affecting >40% of the section |


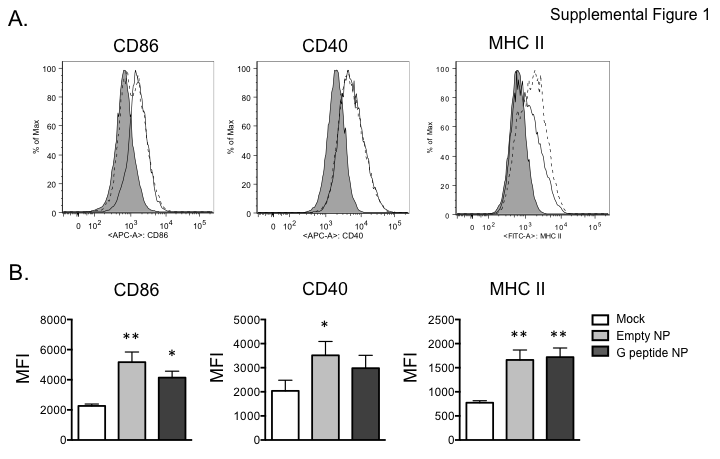


**Supplementary Figure 1. CPTEG:CPH particles activate bovine APC.** Bovine moDC were seeded and stimulated for 24 hours as in Figure 2. The next day, cells were harvested and analyzed by flow cytometry for surface expression of CD86, CD40 and MHC class II. Representative histograms are shown in A for unstimulated moDC (dark grey), moDC stimulated with empty CPTEG:CPH particles (dotted lines) and moDC stimulated with G peptide-loaded CPTEG:CPH particles (solid black lines). The mean fluorescence intensity (MFI) for each marker is presented in B. Data represent n=5 animals per group and are representative of 2 independent experiments. Results in B are presented as means ± SEM. *p<0.05 **p<0.01 compared to mock stimulated cultures.


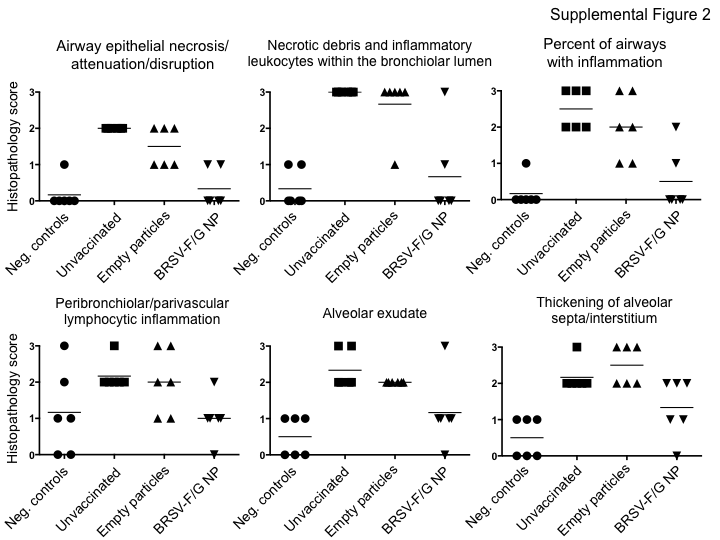


**Supplementary Figure 2. Reduced microscopic pathology in calves receiving the mucosal BRSV-F/G nanovaccine.** Treatment groups included unvaccinated, uninfected negative control calves; unvaccinated calves challenged with BRSV strain 375; calves vaccinated with an ‘empty’ CPTEG:CPH nanovaccine and challenged with BRSV strain 375; and calves vaccinated with the BRSV-F/G CPTEG:CPH nanovaccine and challenged with BRSV strain 375. Animals were euthanized and necropsied on day 7 after challenge. Sections of lung were collected from multiple locations and microscopic lesions were evaluated by a pathologist in a blinded manner. The severity of the lung lesions was scored based upon the six criteria described in Supplemental Table 2. Scores for each animal on each individual criteria are presented. Results represent n=6 animals/group.
